# The Faster-Onset Antidepressant Effects of Hypidone Hydrochloride (YL-0919) in Monkeys Subjected to Chronic Unpredictable Stress

**DOI:** 10.3389/fphar.2020.586879

**Published:** 2020-11-26

**Authors:** Yong-Yu Yin, Chao-Yang Tian, Xin-Xin Fang, Chao Shang, Li-Ming Zhang, Qiang Xu, Yun-Feng Li

**Affiliations:** ^1^ Beijing Institute of Pharmacology and Toxicology, State Key Laboratory of Toxicology and Medical Countermeasures, Beijing Key Laboratory of Neuropsychopharmacology, Beijing, China; ^2^ Hainan Jingang Biotech Co., Ltd., Haikou, China; ^3^ Key Laboratory of Basic Pharmacology of Ministry of Education and Joint International Research Laboratory of Ethnomedicine of Ministry of Education, Zunyi Medical University, Zunyi, China; ^4^ Institute of Military Veterinary Medicine, Academy of Military Medical Science, Changchun, China; ^5^ Yantai Yuhuangding Hospital, Yantai, China; ^6^ Beijing Institute of Basic Medical Sciences, Beijing, China

**Keywords:** hypidone hydrochloride (YL-0919), faster-onset, antidepressants, chronic unpredictable stress, monkeys

## Abstract

Given the limited monkey models of depression available to date, as well as the procedural complexity and time investments that they involve, the ability to test the efficacy and time course of antidepressants in monkey models is greatly restricted. The present study attempted to build a simple and feasible monkey model of depression with chronic unpredictable stress (CUS) and evaluate the antidepressant effect and onset time of fluoxetine hydrochloride (FLX) and the new drug hypidone hydrochloride (YL-0919), a potent and selective 5-HT reuptake inhibitor, 5-HT_1A_ receptor partial agonist and 5-HT_6_ receptor full agonist. Female cynomolgus monkeys with low social status in their colonies were selected and subjected to CUS for 8 weeks by means of food and water deprivation, space restriction, loud noise, strobe light, and intimidation with fake snakes. Huddling, self-clasping, locomotion and environmental exploration were monitored to evaluate behavioral changes. In addition, the window-opening test was used to evaluate the exploratory interest of the monkeys. The present results revealed that CUS-exposed monkeys displayed significant depression-like behaviors, including significant decreases in exploratory interest, locomotion, and exploration as well as significant increases in huddling and self-clasping behavior and the level of fecal cortisol after 8 weeks of CUS. Treatment with FLX (2.4 mg/kg, i. g.) or YL-0919 (1.2 mg/kg, i. g.) markedly reversed the depression-like behaviors caused by CUS, producing significant antidepressant effects. YL-0919 (once daily for 9 days) had a faster-onset antidepressant effect, compared with FLX (once daily for 17 days). In summary, the present study first established a CUS model using female cynomolgus monkeys with low social status and then successfully evaluated the onset time of 5-HTergic antidepressants. The results suggested that monkeys exposed to CUS displayed significant depression-like behaviors, and both FLX and YL-0919 produced antidepressant effects in this model. Moreover, YL-0919 appeared to act faster than FLX. The present study provides a promising prospect for the evaluation of fast-onset antidepressant drugs based on a CUS monkey model.

## Introduction

Depression is a ubiquitous mental disorder and affects approximately 322 million people worldwide, and it is predicted that depression will rise to the top of the world’s disease burden by 2030 (WHO, 2017). Existing first-line antidepressants, such as selective serotonin reuptake inhibitors (SSRIs), serotonin and norepinephrine reuptake inhibitors (SNRIs) are the main treatment for depression. However, these antidepressants have some deficiencies, such as delayed onset of action, low effective rate, cognitive impairment and so on. Therefore, the development of new antidepressants with rapid onset and low adverse reactions has become a global hot focus.

It is well known that animal models are critical to the development of antidepressants. As early as the beginning of this century, the development of better animal models was recommended as one of the highest priorities for the National Institute of Mental Health (NIMH) in depression research areas (Nestler et al., 2002). Rodent models have been extensively used in preclinical studies on existing antidepressants, but rodents cannot always accurately model human behavioral and biological responses (Seok et al., 2013). In particular, the differences between rodents and humans, such as brain structures (Herculano-Houzel et al., 2006) and cell types (Hodge et al., 2019), make many potential drugs work well on animals in preclinical studies but not on humans in clinical studies (van der Worp et al., 2010; Hartung, 2013; Perrin, 2014). As a result, the excessive use of rodent models might limit the discovery and evaluation efficiency of new drugs, which has also become one of the important reasons for the failure of the development of new antidepressants in recent years (Perrin, 2014). Unlike rodents, nonhuman primates, such as monkeys, are highly similar to humans in many aspects (Fujiyama et al., 2002; Lu et al., 2008; Phillips et al., 2014), and they especially have abundant higher affective activity. Consequently, monkey models of depression can better mimic core symptoms of depression than rodents, which can play a vital role in bridging the gap between basic research and clinical research.

To our knowledge, the main monkey models of depression mentioned in previous reports were as followed. Harlow & Suomi started to use a visual plus social isolation model by blocking visual communication and social interaction between subjects and other monkeys (Harlow and Suomi, 1971; Suomi and Harlow, 1972). Variations of social isolation model, which separated monkeys into single-cages for several months, were reported in subsequent studies (Harlow and Suomi, 1974; Suomi et al., 1975; Li et al., 2013). Researchers also established maternal separation model, in which monkeys displayed significant depression-like behaviors after half a year of separation from their mothers (Feng et al., 2011). In addition, it was reported that short photoperiod conditions could lead monkeys to display huddling behavior and a winter depression model was firstly set up (Qin et al., 2015). Studies reported that macaques exhibited a naturally-occurring depression similar to humans (Xu et al., 2015), and postpartum macaques could exhibit a natural model of behavioral depression (Chu et al., 2014).

These monkey models of depression mentioned above might provide an important research platform for the mechanism of depression and the development of antidepressants. The huddling behavior noted in the above models is the core behavioral characteristic in current monkey models of depression, providing an important reference for the evaluation of depression-like behaviors in monkeys. However, the shortcomings of these models, including complicated procedures, long modelling times and high rates of failure, cannot be ignored (Feng et al., 2011; Li et al., 2013; Xu et al., 2015), which greatly limits preclinical studies of depression and the efficiency of new antidepressant discovery. To date, there have been no reports on the onset time evaluation of antidepressants in monkey models of depression. Taken together, the literature suggests that establishing simple and efficient monkey models may be of great significance for evaluating the effect and onset time of new antidepressants.

Hypidone hydrochloride (YL-0919) is a potent triple SSRI, 5-HT_1A_ partial agonist and 5-HT_6_ full agonist developed by our institute, which has entered into the phase II clinical trial. Our previous studies revealed that YL-0919 could produce significant antidepressant effects in the sucrose preference test (SPT), the forced swimming test (FST) and the novelty suppressed feeding test (NSF) in CUS-exposed Wistar rats (Ran et al., 2018; Sun et al., 2019). Furthermore, compared with FLX (fluoxetine, SSRI), YL-0919 exerted a faster-onset antidepressant effect and enhanced cognitive function in Morris water maze test (Chen et al., 2018) without sexual dysfunction (Zhang et al., 2017) in ICR mice. The 5-HT_1A_ receptor partial agonist and 5-HT_6_ receptor full agonist, the targets of YL-0919, may be the underlying mechanism of the faster onset and enhanced cognitive function, respectively.

The present study first applied CUS to adult female cynomolgus monkeys with low social status and explored the effects of CUS on a series of behaviors. The window-opening test was used to evaluate the exploratory interest of the monkeys. Furthermore, we then identify the onset time of YL-0919, and FLX was used as a parallel control to evaluate the reliability of the model. The present study aimed to establish a simpler, reliable and short-term monkey model of depression for the first time and provided an experimental basis for the evaluation of faster-onset antidepressants.

## Materials and Methods

### Animals

A total of 16 adult female cynomolgus monkeys weighing 3.4–4.5 kg and aged 32–40 months were provided by Hainan Jingang Biotech Co., China. After 1 month of continuous observation, the 10 monkeys with low social status, which tended to be alone, always bullied by other monkeys and afraid to fight for food with other monkeys, served as the stress group, and the other six normal monkeys served as the control group. Each monkey in the present study was singly housed in the cage (cage unit: 70.0 × 60.0 × 80.0 cm) for 4 weeks to acclimatize to the laboratory environment with room temperature (22–28°C), humidity (45–70%), and a 12 h light/12 h dark cycle (lights on at 07:30 AM). At the end of CUS, the 10 monkeys subjected to CUS were divided into two groups that were intragastrically administered FLX and YL-0919. In addition to the necessary stressors that the stress monkeys underwent, including water deprivation, food deprivation, and space restriction, all moneys during the study were provided with compound monkey food and fresh fruits daily, and access to tap water ad libitum.

### Ethics Declaration

All procedures were in strict accordance with the guidelines of the National Institutes of Health Guide for the Care and Use of Laboratory Animals (NIH Publications No. 80-23, revised in 1996) and the animal study was reviewed and approved by the National Animal Research Authority (China) and Beijing Institute of Pharmacology and Toxicology. All efforts were made to reduce the number of monkeys used and to minimize animal suffering.

### Drugs and Dose

YL-0919 (purity: 99.9%) was provided by the drug synthesis laboratory of the Institute of Pharmacology and Toxicology. FLX (product number: 20170201) was purchased from Jiangsu Changzhou Siyao Pharmaceutical Co., Ltd (China). Previous studies showed that the effective antidepressant dose of YL-0919 in rodent models ranged from 0.625 to 5 mg/kg, and 2.5 mg/kg could exert a better antidepressant effect (Zhang et al., 2017; Chen et al., 2018; Ran et al., 2018; Sun et al., 2019). According to body surface area (Pinkel, 1958; Felici et al., 2002), it was calculated that the effective dose of YL-0919 in monkeys could be 1.2 mg/kg. It was reported that 2.4 mg/kg was the effective dose of FLX in monkeys (Golub et al., 2016). In addition, FLX (2.4 mg/kg) in monkeys could provide similar pharmacokinetic measures as humans taking 20 mg/day (Shrestha et al., 2014). Thus, FLX (2.4 mg/kg) was selected in the present study. All drugs used in the experiment were dissolved in saline and administered intragastrically in a volume of 4 ml/kg.

### Chronic Unpredictable Stress

**FIGURE 1:**
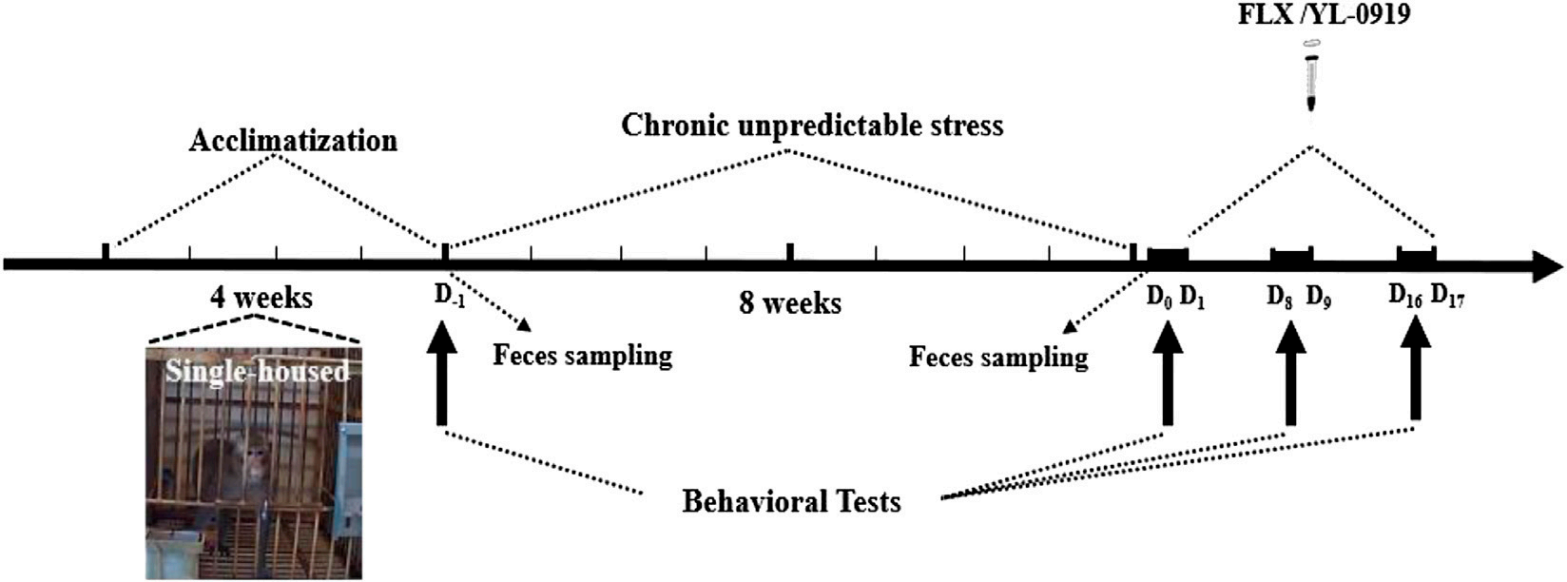
The CUS and behavioral experiment paradigm. All monkeys were singly housed for acclimatization before CUS. The 10 CUS monkeys were treated daily with two different stressors, while the six normal monkeys served as controls without any stressors. Before CUS, behavioral data and fecal samples were collected at D_-1_. After CUS, behavioral data and fecal samples were collected at D_0,_ and the window-opening test was performed at D_1_. Then, 10 CUS monkeys were divided into two groups, with five monkeys in each group. The two groups were intragastrically administered FLX (2.4 mg/kg) or YL-0919 (1.2 mg/kg). Drug treatment began at D_1_ and ended at D_17_. Behavioral video recording was performed at D_9_ and D_17_, and the window-opening test was performed at D_8_ and D_16_.

In the present study, the monkeys underwent 8 weeks of CUS exposure (see Table 1), each following the same schedule as the first week. The main stressors included restraint, intimidation, food deprivation, water deprivation, strobe light, and loud noises. The present study used fake snakes of various colors to intimidate monkeys and reduced the space of the cage with one push-pull device to restrain monkeys. Two stressors were applied each day, and each stressor generally lasted 12 h. During the period of CUS, each monkey in the control group was singly housed into similar environment with the stress monkeys, including temperature, humidity and so on. Furthermore, the control monkeys underwent without any stressors, access to food and tap water ad libitum. The detailed experimental procedures are shown in Figure 1.

**TABLE 1:** The stressors and routines of chronic unpredictable stress in the first week.

Day	Stressor 1 (Day 07:30–19:30)	Stressor 2 (Night 19:30–07:30)
Monday	Food deprivation	Strobe light
Tuesday	Water deprivation	Loud noise
Wednesday	Intimidation	Space restriction
Thursday	Loud noise	Food deprivation
Friday	Space restriction	Strobe light
Saturday	Intimidation	Loud noise
Sunday	Water deprivation	Space restriction

### Behavioral Categories

To record the behaviors of monkeys, two 5-min videos of each monkey were recorded at 9:30–11:30 AM and 1:00–3:00 PM. The present study employed four behavioral categories (see Table 2) defined by previous studies to assess the behaviors of monkeys (Harlow and Suomi, 1971; McKinney et al., 1972; Suomi and Harlow, 1972; Suomi et al., 1978). In the present study, huddling and self-clasping were categorized as negative behaviors, and locomotion and exploration were categorized as positive behaviors. One skilled and trained technician scored the behaviors employing a modified frequency scoring system previously defined (Harlow and Suomi, 1974; Moran and McKinney, 1975; Suomi et al., 1975), and behaviors of monkeys falling within each of four behavioral categories mentioned below were recorded for presence or absence during each of the 20 15 s intervals comprising every 5 min observing session.

**TABLE 2:** Description of behavioral categories applied.

Categories	Detailed description
Huddling	Self-enclosed, fetal-like position incorporating any or all patterns of self-clasping, self-embracing, or a lowered head, all of which may be accompanied by repetitive movements
Self-clasping	Clasping of any part of own body with the hand and/or foot
Locomotion	Ambulation of one or more full steps
Exploration	Touching and/or manipulation by subject of inanimate objects, e.g., towel, toy, or cage lock

### Window-Opening Test

**FIGURE 2:**
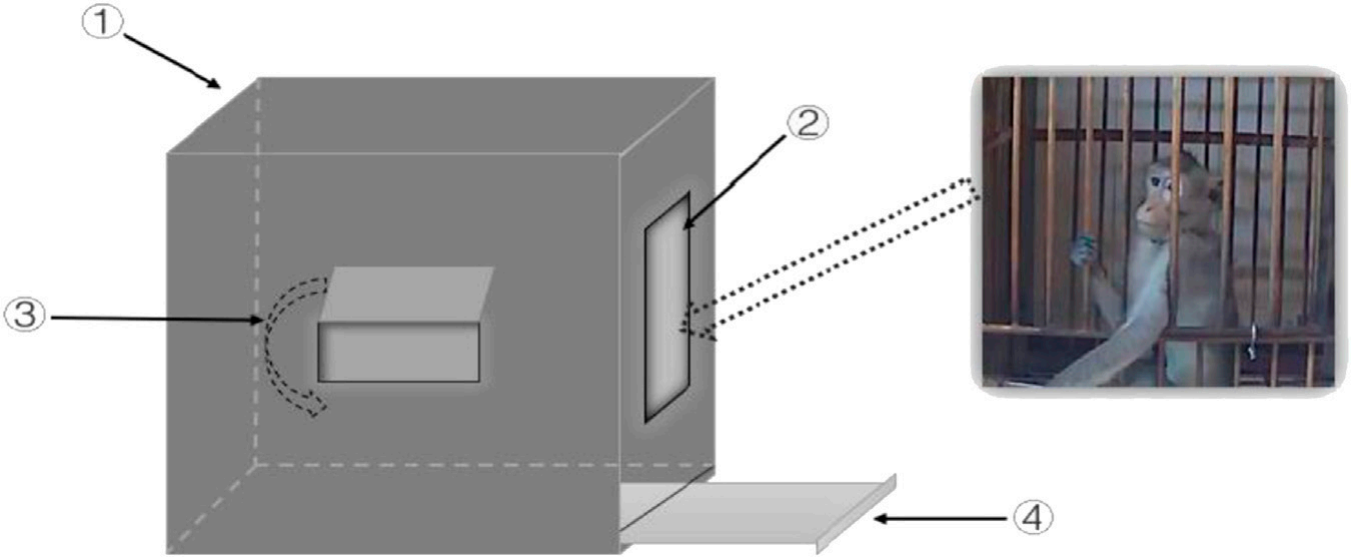
Schematic diagram of the window-opening test device. ① The wooden box with specifications of 50 × 50 × 50 cm (length × width × height); ② The gate 20 × 20 cm (length × width) was closed during the experiment, through which the monkey could be moved in or out; ③ The small window 5 × 10 cm (length × width) that the monkeys could push out with the limbs or the head; ④ The excrement collection box that was used to receive the monkey’s urine and feces was cleaned after each experiment to reduce the influence of fecal odor on the behaviors of monkeys.

The present study first used the window-opening test to evaluate the exploratory interest of monkeys. The monkeys were removed from their single cages and placed in the test device by a professional keeper. The monkeys were kept in the device for 15 min, and their behavior was recorded with cameras. The latency of the monkeys to open the small window and the total duration that the monkeys spent with the small window open were recorded. The test device and the detailed procedures are shown in Figure 2.

### Feces Samples

The fresh feces of monkeys were collected at 9:00 to 10:00 AM and stored at −80°C until assayed. The fecal cortisol extraction procedures were described in a previous study (Terio et al., 2002), and the present study tested the cortisol level by using a monkey cortisol ELISA purchased from Shanghai Fusheng Industrial Co. Ltd. (Shanghai, China). The assays were performed following the manufacturer’s protocol. The optical density (OD value) was measured immediately using a microplate reader at 450 nm.

### Statistical Analysis

No data was excluded in the present study, and all data were expressed as the mean ± SEM and were analyzed with GraphPad Prism 8.0 software. The differences between pre-stress and post-stress in control group or stress group were analyzed with paired-samples *t* test, and the differences of post-stress between control group and stress group were analyzed with Student’s *t* test. The differences between FLX group or YL-0919 group were analyzed with two-way ANOVA, and if the main effect was significant, Dunnett’s test was needed. For all tests, *p* < 0.05 was considered to be statistically significant.

## Results

### Effects of Chronic Unpredictable Stress on the Behaviors and Fecal Cortisol of Monkeys

**FIGURE 3:**
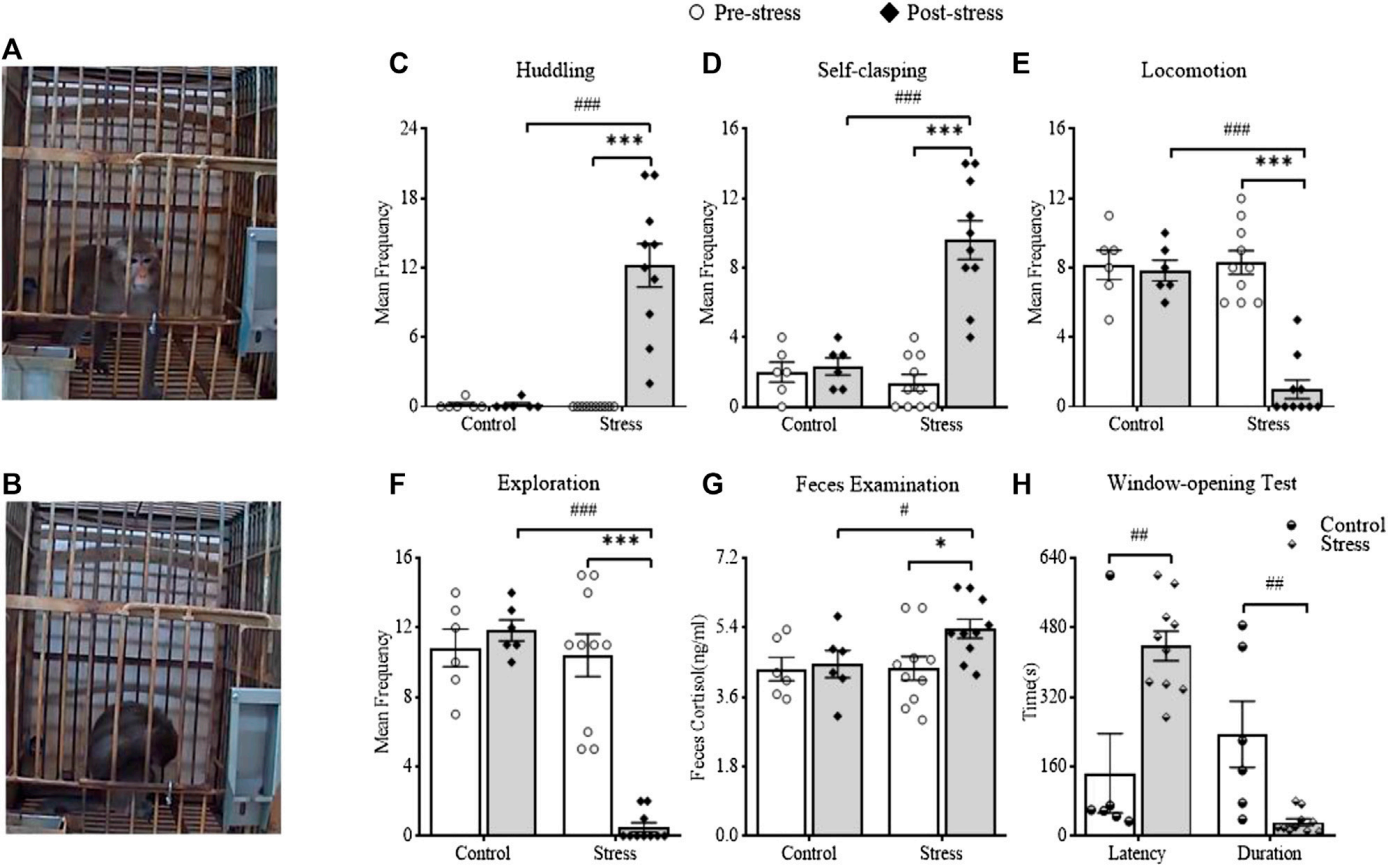
The effects of CUS on behaviors, feces cortisol and the exploratory interest of monkey. A showed the monkey displayed normal behavior before CUS. B showed the monkey displayed huddling behavior after CUS. C and D showed changes in negative behaviors between pre-stress and post-stress, including huddling and self-clasping. E and F showed changes in positive behaviors between pre-stress and post-stress, including locomotion and exploration. G showed the changes in fecal cortisol between pre-stress and post-stress. H showed the latency and duration of monkeys in the window-opening test. ^#,^*p < 0.05, ^##,^**p < 0.01, ^###,^***p < 0.001, vs control or pre-stress (*n* = 6 or 10/group).

Twenty-four hours after the last stressor, negative and positive behaviors were recorded. The results showed that huddling behavior (as illustrated in Figure 3B) (*t*_(9)_ = 6.494, *p* = 0.0001, vs pre-stress of the stress group; *t*_(14)_ = 4.886, *p* = 0.0002, vs post-stress of the control group) and self-clasping behavior (*t*_(9)_ = 6.318, *p* = 0.0001, vs pre-stress of the stress group; *t*_(14)_ = 4.852, *p* = 0.0003, vs post-stress of the control group) significantly increased (as illustrated in Figures 3C,D). Meanwhile, locomotion behavior (*t*_(9)_ = 8.156, *p* < 0.0001, vs pre-stress of the stress group; *t*_(14)_ = 8.158, *p* < 0.0001, vs post-stress of the control group) and exploration (*t*_(9)_ = 8.472, *p* < 0.0001, vs pre-stress of the stress group; *t*_(14)_ = 19.72, *p* < 0.0001, vs post-stress of the control group) significantly reduced (as illustrated in Figures 3E,F). The above results suggested that CUS had significant effects on both the negative and positive behaviors of monkeys and that the monkeys displayed significant depression-like behavior. The window-opening test was the first to be designed and applied to evaluate the exploratory interest of monkeys in the present study. The latency was significantly longer (*t*_(14)_ = 3.563, *p* = 0.0031, vs control, independent-sample *t*-test), but the duration was significantly shorter (*t*_(14)_ = 3.459, *p* = 0.0038, vs control) in the CUS-exposed monkeys (as illustrated in Figure 3H), which might indicate that CUS could significantly decrease the exploratory interest of monkeys.

To assess the effect of CUS on the fecal cortisol of monkeys, we performed ELISAs (as illustrated in Figure 3G). The results suggested that the post-stress monkeys exhibited significantly elevated levels of cortisol (*t*_(9)_ = 3.122, *p* = 0.0123, vs pre-stress of the stress group; *t*_(14)_ = 2.164, *p* = 0.0482, vs post-stress of the control group), which suggested that CUS could remarkedly increase the function of the hypothalamic–pituitary–adrenal (HPA) axis in the CUS-exposed monkeys.

### Effects of Fluoxetine Hydrochloride or YL-0919 on Depression-Like Behaviors in Chronic Unpredictable Stress-Exposed Monkeys

**FIGURE 4:**
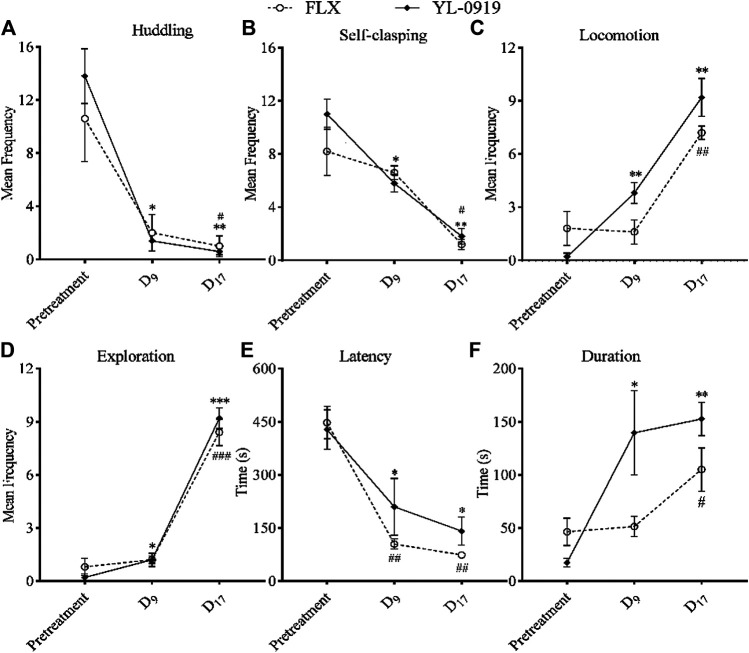
Effects of FLX (2.4 mg/kg, i. g) or YL-0919 (1.2 mg/kg, i. g) on depression-like behaviors in CUS-exposed monkeys. After 8 weeks of CUS, the CUS-exposed monkeys were divided into two groups and were intragastrically administered FLX or YL-0919, respectively. **(A, B)** showed the effects of FLX or YL-0919 on huddling and self-clasping behavior, respectively. **(C, D)** showed the effects of FLX or YL-0919 on locomotion and environmental exploration, respectively. E and F showed the effects of FLX or YL-0919 on latency and duration in the window-opening test, respectively. For FLX or YL-0919 group, ^#,^ *p < 0.05, ^##,^ **p < 0.01, ^###,^ ***p < 0.001, vs pretreatment (*n* = 5/group).

Treatment with FLX once daily for 17 days (*p* = 0.0364) or with YL-0919 once daily for just 9 days (*p* = 0.0101) significantly reduced huddling (Figure 4A, F_(2,16)_ = 35.37, *p* < 0.0001, two-way ANOVA followed by Dunnett’s test), and treatment with FLX for 17 days (*p* = 0.0488) or with YL-0919 for just 9 days (*p* = 0.01) also significantly reduced self-clasping (Figure 4B, F_(2,16)_ = 30.78, *p* = 0.0001) in the CUS-exposed monkeys. In addition, treatment with FLX for 17 days (*p* = 0.0034) or with YL-0919 for just 9 days (*p* = 0.0037) significantly increased locomotion (Figure 4C, F_(2,16)_ = 94.95, *p* < 0.0001), and treatment with FLX for 17 days (*p* = 0.0009) or with YL-0919 for just 9 days (*p* = 0.0421) also significantly increased exploration (Figure 4D, F_(2,16)_ = 273.4, *p* < 0.0001) in the CUS-exposed monkeys. In the window-opening test, treatment with FLX (*p* = 0.0037) or YL-0919 (*p* = 0.0235) once daily for just 9 days both significantly decreased latency (Figure 4E, F_(2,16)_ = 49.88, *p* < 0.0001), and treatment with FLX for 17 days (*p* = 0.0253) or with YL-0919 for 9 days (*p* = 0.0494) significantly increased duration (Figure 4F, F_(2,16)_ = 14.39, *p* = 0.0023) in the CUS-exposed monkeys. These results indicated that treatment with YL-0919 once daily for 9 days could significantly reverse the depression-like behaviors caused by CUS and exert a faster-onset antidepressant effect on this model, compared with FLX (once daily for 17 days).

## Discussion

In the present study, the model was built for the first time with CUS procedures in monkeys with low social status, which could mimic a series of negative and positive depression-like behaviors in monkeys, especially huddling behavior, which is the core symptom of depression in monkey models. Furthermore, the window-opening test was established to evaluate the exploratory interest of monkeys, and CUS significantly reduced the exploratory interest of monkeys. Eventually, the results further demonstrated that treatment with FLX or YL-0919 could exert significant antidepressant effects on this model, and YL-0919 might exert a faster-onset antidepressant effect compared with FLX.

Depression is regarded as the result of interactions between multiple genes and the environment (Ball et al., 2009; Koenen and Galea, 2009; Rieckmann et al., 2009). To better mimic the symptoms of clinical depression, monkeys with low social status were first used to build the CUS model in the present study. These monkeys could be prone to depression (Murphy et al., 1991; Qin, et al., 2015), which may reflect the potential genetic background of depression. Furthermore, these monkeys were more susceptible to assaults from other monkeys and more sensitive to stress (Shively et al., 1997). The present study demonstrated that 8 weeks of CUS could lead to a series of depression-like behaviors in monkeys, including huddling behavior and loss of exploratory interest. In fact, loss of interest is the core symptom of depression (Association, 2000), and sucrose preference is usually used as an indicator of anhedonia in rodents subjected to CUS (Markov et al., 2017), which has been widely applied for the evaluation of the efficacy and onset time of antidepressants (Liu et al., 2018). The window-opening test first used in the present study reflected the exploratory interest. Interestingly, the results demonstrated that CUS could significantly reduce the exploratory interest of monkeys, and treatment with FLX for 17 days or treatment with YL-0919 for 9 days significantly increased the exploratory interest of monkeys, displaying a significant antidepressant effect, which indicated that the window-opening test could well mimic the loss of interest. Herein, these results suggested that the model might have a good face validity.

The model in the present study not only overcame the disadvantages of other monkey models of depression but also might have more advantages such as simpler operation and shorter modelling period, as well as being more controllable for modeling process (Feng et al., 2011; Li et al., 2013; Xu et al., 2015), which further enhanced the evaluation efficiency of the antidepressant effect and could be used for preclinical studies of the mechanism of depression. In addition, previous studies have revealed that patients with depression display behaviors similar to huddling behavior (Harlow and Suomi, 1974; Canales et al., 2010), which has become one of the core symptoms of depression in monkeys (Harlow and Suomi, 1971; Shively et al., 2005). This behavior is usually used to evaluate the depression level of monkeys, which means that the more huddling monkeys display, the higher level of depression monkeys exhibit. The data from the present study suggested that the huddling behavior in CUS-exposed monkeys significantly increased, which was consistent with the results of previous studies (Zhang et al., 2016).

To date, there have been no reports about the onset time course of antidepressants in monkey models of depression, and the present study first evaluated the onset speed of antidepressants in CUS-exposed monkeys. SSRIs usually took 2–6 weeks to response clinically. However, treatment with YL-0919 (i.g.) for about 3–7 days could produce antidepressant behavior effects and increase the long-term potentiation of the hippocampus in rats (Zhang et al., 2017; Ran et al., 2018; Sun et al., 2019). Therefore, for reflecting the difference of onset speed between YL-0919 and FLX, as well as minimizing the disturbance of repeated catching on monkeys’ exploratory behavior as well as other behavioral indicators, we evaluated the behavioral indicators of monkeys only at D_9_ and D_17_. The current results showed that treatment with FLX for 17 days reversed the depression-like behavior caused by CUS, including significantly decreasing huddling and self-clasping, as well as significantly increasing locomotion and exploration. In other words, FLX exerted a significant antidepressant effect in CUS-exposed monkeys, which was consistent with the onset time course of FLX in the clinic (Tollefson et al., 1995). The results above showed that the model in the present study might have a good predictive validity.

Herein, we also investigated the onset time of YL-0919 in this model. The results suggested that treatment with YL-0919 once daily for 9 days could significantly decrease huddling and self-clasping as well as increase locomotion and exploration. These findings were similar to our previous studies which revealed that treatment with YL-0919 for 3–7 days could produce an antidepressant effect in CUS-exposed rats (Ran et al., 2018). Our previous behavioral study found that YL-0919 or vilazodone (a dual 5-HT_1A_ partial agonist and SSRI) exerted faster antidepressant actions (4 days in the SPT and 6 days in the NSFT). However, FLX (SSRI) took 20–22 days to exert significant antidepressant action under the same experimental conditions (Sun et al., 2019). In addition, treatment with YL-0919 for 7 days increased the long-term potentiation (LTP) of the hippocampus in rats, but it took 21 days for FLX to enhance the hippocampal LTP under the same experimental conditions (Zhang et al., 2017). These findings emphasized the importance of 5-HT_1A_ receptor in the faster-onset antidepressant effect of YL-0919. Additional data are warranted to determine the contribution of the 5-HT_6_ receptor antagonism of YL-0919, a pharmacological property that has been pre-clinically shown to have antidepressant-like properties (Zajdel et al., 2016; Millan et al., 2020). Furthermore, the dosage range of YL-0919 in this animal model was similar to that in a rat model of chronic unpredictable stress (Ran et al., 2018; Sun et al., 2019), which might indicate that YL-0919 could have a faster-onset antidepressant effect in the monkey model of depression. Collectively, our findings in rodents and monkeys indicate that YL-0919 has a fast onset antidepressant effect and these data are pending a clinical validation (ongoing clinical trial in a small cohort of 45 depressed patients receiving a daily oral dose of 20 mg YL-0919).

The present study wanted to explore the effects of CUS or antidepressants on these behaviors and used the self-controlled study before and after treatment to reduce individual differences. Generally, there were actually some behavioral differences for each monkey before 8 weeks CUS in the present study, and these behavioral differences also existed in other normal monkeys and were normal attributes of monkeys. In addition, the purpose of the present study was to build the monkey model of depression and then evaluate the effects of antidepressants on this model, and further studies about the effects of antidepressants on this monkey model of depression are needed in the future.

In summary, the model used in the present study was successfully built for the first time with CUS procedures in monkeys with low social status. The model had a simpler operation and shorter modelling period than other monkey models of depression and could effectively mimic the core symptoms of depression, which provided a better animal model and research idea for the preclinical study of the pathogenesis of depression, the screening of antidepressants, and the effect and onset speed of antidepressants. Moreover, the present study further clarified the faster-onset antidepressant effect of YL-0919 and provided a good reference for research on antidepressants.

## Data Availability Statement

The raw data supporting the conclusion of this article will be made available by the authors, without undue reservation.

## Ethics Statement

The animal study was reviewed and approved by the National Animal Research Authority (China) and Beijing Institute of Pharmacology and Toxicology.

## Author Contributions

Y-YY performed the research design, the behavioral tests, data analysis and wrote the manuscript. C-YT contributed to the behavioral tests. X-XF and CS provided some help in the behavioral tests and ELISA. L-MZ contributed to the writing and revising of the manuscript. QX and Y-FL contributed to the research design and revised the manuscript. All authors approved the final manuscript.

## Funding

This work was supported by National Natural Science Foundation of China (No. 81773703) and the National Key New Drug Creation Program of China (No. 2017ZX09309012).

## Conflict of Interest

Author C-YT was employed by the company Hainan Jingang Biotech Co., Ltd.The remaining authors declare that the research was conducted in the absence of any commercial or financial relationships that could be construed as a potential conflict of interest.
